# Web-Based Interventions to Promote Healthy Lifestyles for Older Adults: Protocol for a Scoping Review

**DOI:** 10.2196/23207

**Published:** 2021-01-04

**Authors:** Audrey Lavoie, Véronique Dubé

**Affiliations:** 1 Research Center Centre hospitalier de l’Université de Montréal Montreal, QC Canada; 2 Faculty of Nursing Université de Montréal Montreal, QC Canada

**Keywords:** Aged, web-based intervention, healthy lifestyle, behavioral change, components, effects

## Abstract

**Background:**

With the aging of the population and rising rates of chronic diseases, older adults need support if they are to adopt healthy lifestyles. Web-based interventions should be considered for this purpose, since they are easily accessed and can foster healthy lifestyles among older adults. However, the literature on such interventions discusses a variety of components and effects and provides only 2 syntheses of knowledge on web-based interventions with older adults. These studies focus on populations aged 50 years and older, whereas the components and effects of interventions for a population of older adults (ie, 65 years and older) may differ. In addition, these 2 syntheses examined only quantitative studies, although other types of studies (ie, qualitative) are available and could help advance knowledge in this field. A scoping review is therefore relevant in order to explore the extent of the literature on this subject.

**Objective:**

The purpose of the study described by this protocol is to explore the extent of the literature (experimental, quasi-experimental, qualitative, systematic reviews, and grey literature) on the components and effects of web-based interventions as a way to promote healthy lifestyles among older adults.

**Methods:**

The databases MEDLINE, CINAHL, PsycInfo, Web of Science, Cochrane Database of Systematic Review and Joanna Briggs Library will be searched, in addition to the grey literature using Google Scholar and OpenGrey. Studies will be selected for the review by 2 researchers, working independently. The data will be synthesized based on the conceptualization of web-based interventions (ie, behavior change techniques, dispensation modes, and theories). A thematic analysis will be performed to summarize the components of the interventions studied.

**Results:**

The database search will begin in August 2020 and be completed in October 2020.

**Conclusions:**

This scoping review should highlight web-based interventions designed to promote healthy lifestyles, as well as their components and effects, among people aged 65 years and older. These results could provide important guidance for intervention developers and designers in identifying the components of web-based interventions relevant to older adults and lead to further studies on this topic.

**International Registered Report Identifier (IRRID):**

PRR1-10.2196/23207

## Introduction

The number of older adults worldwide (ie, those aged 65 years and older) is expected to almost double over the next 30 years, from 12% to 22% [1]. This significant aging of the population will not be without consequences for health care systems. In fact, this phenomenon will lead to an increase in the rate of chronic diseases, considering that the prevalence of these diseases increases with age and that older adults are seriously affected by them [2,3]. In addition to having beneficial effects on health, healthy lifestyle habits (healthy eating, regular physical activity, smoking abstinence, limiting alcohol consumption, management of stress) could help prevent a significant number of diseases [2] and promote longevity [4]. However, healthy lifestyle adoption is a complex phenomenon, especially in a population of older adults whose behaviors may have been strongly entrenched for many years. Among other things, lack of motivation is one of the greatest barriers to older adults' to adopting a healthy lifestyle [5-7]. This is why they should be able to benefit from interventions aimed at bolstering their motivation to change.

Moreover, with advances in health technologies, web-based is increasingly the preferred method of intervention, even among older adults, for whom internet use has been growing rapidly in recent years [8]. Web-based interventions could be used to support individuals as they adopt healthy lifestyles and would be favorable to older adults [9]. In addition, they constitute an economical and accessible alternative for the health care system [10]. Web-based interventions are all the more relevant in the context of the current global pandemic, since the modes of intervention delivery need to be reconsidered to limit face-to-face contact. The recommendations on social distancing must be followed in order to preserve the population’s health, especially among vulnerable older adults. In this context, lifestyle habits can also be disrupted (eg, sedentary behavior, dietary changes) [11], and older adults’ access to programs and services that facilitate the adoption of healthy lifestyles (eg, gyms) is limited [12]. As a result, web-based interventions may be a solution to help individuals adopt and maintain healthy lifestyles [12].

However, although the literature documents numerous web-based interventions, their components and effects are diverse, making it difficult to draw conclusions about which components lead to optimal change outcomes for this population. In this regard, Webb et al [13] developed a framework to facilitate investigations of the components of web-based interventions that will optimally influence behavior change. They propose categorizing the various intervention components according to the behavior change techniques (eg, problem solving, barrier identification, information), delivery modes (eg, interaction, feedback, additional modes), and theories (eg, Theory of Planned Behavior, Social Cognitive Theory, Health Belief Model) used [13].

While some authors have published syntheses of the literature focused on web-based interventions designed to promote lifestyle changes in adults [9,13,14], to our knowledge, no synthesis of knowledge has focused on people aged 65 years and older. In fact, there appears to have been only 2 systematic reviews [15,16] conducted on web-based interventions aimed at healthy lifestyle habits for people aged 50 years and older. The primary studies included in these systematic reviews have small sample sizes with an average age of 50 years or older, which means that they do not focus specifically on a population of older adults [15,16]. Although it is difficult to reach a consensus on the specific age threshold used in the literature to define old age, the World Health Organization [2] suggests defining older adults as persons aged 65 years and older. Because older adults are a heterogeneous group with multiple characteristics, older individuals, such as those aged 65 years and older, may have different needs to be met by these interventions because of the biological (eg, decreased functional capacity, frailty) and psychological changes (eg, changes in roles, social position and priorities) associated with aging [2]. Moreover, older persons should be able to benefit from accessible health services adapted to their needs [17]. Since the components of the interventions as well as their effects could differ for this population, it is essential to explore the literature on this subject.

Finally, these 2 syntheses of the literature [15,16] focus only on quantitative studies, whereas qualitative studies are also available in the literature. Both qualitative and quantitative studies can make relevant contributions on the components and effects of web-based interventions to promote healthy lifestyles among older adults. Qualitative studies could also greatly enrich our knowledge on the impact of these interventions on older adults. A scoping review on this topic is appropriate since, to our knowledge, no study has explored the extent of knowledge on web-based interventions for people aged 65 years and older, including both qualitative and quantitative studies.

Therefore, the purpose of this article is to present the protocol for a scoping review that will explore the extent of the available literature on web-based interventions aimed at promoting healthy lifestyles among people aged 65 years and older.

## Methods

### Overview

This protocol describes the methodology that will be used to conduct a scoping review and the steps that will be followed to ensure its rigor. A scoping review will be conducted based on the framework proposed by Levac et al [18]. This framework was initially developed by Arksey and O'Malley [19] and then refined by Levac et al [18] at the level of its methodological description, which is why it has been chosen. According to Arksey and O'Malley [19], a scoping review may be conducted to determine the scope of the research or to map the available literature on a phenomenon, which is the purpose of this study. The review will follow the 6 steps proposed by Levac et al [18] as presented below.

### Identifying the Research Questions

The research questions were identified following a brief review of the initial literature and discussions with the research team, composed of a doctoral student, a researcher, a research professional, and a librarian. This scoping review will seek to answer the following questions: (1) What web-based interventions are aimed at promoting healthy lifestyles among people aged 65 years and older? (2) What are the components of these interventions (ie, behavior change techniques, delivery modes, and theories used)? and (3) What have been the effects of these interventions as reported in the literature?

### Identifying Relevant Studies

The following databases will be searched: MEDLINE, CINAHL, PsycInfo, Web of Science, Cochrane Database of Systematic Review, and Joanna Briggs Library. These databases were selected for their focus on the field of social and health sciences, which is related to the topic of this study. Grey literature will be searched using the Google Scholar and OpenGrey databases. The reference lists of the identified articles will be checked to ensure that all relevant articles have been included. The authors of primary studies will be contacted if additional information is required.

The search strategy, established with a librarian’s assistance, will use keywords and descriptors related to the concepts of older persons (eg, *aged*, *elderly*), lifestyle (eg, *lifestyle*, *exercise)*, and web-based interventions (eg, *internet*, *online*). Multimedia Appendix 1 presents an example of such a strategy. Given the broad nature of a scoping review, the criteria for inclusion will be (1) articles published between 1990 and 2020, since the World Wide Web was created in 1989 [20], (2) articles published in French or English, (3) articles related to the goal of the scoping review (ie, to a web-based intervention addressed to a population of older adults) and aimed at promoting healthy lifestyle habits (ie, healthy eating, regular physical activity, smoking abstention, limiting alcohol consumption, management of stress), and (4) primary studies (eg, experimental, quasi-experimental and qualitative), systematic reviews of any type, and other documents associated with the grey literature (eg, government reports and clinical practice guidelines). The exclusion criteria will be (1) articles in which persons aged 65 years and older are not the population specifically studied, (2) articles in which the web component of the intervention is not predominant (eg, face-to-face interventions complemented by use of a website) because this could mean that the results are not specifically related to the web-based intervention, and (3) articles that do not primarily target healthy lifestyle habits (eg, symptom self-management programs that include physical exercise). Finally, the articles identified will be exported to a data management software program (Covidence, Veritas Health Innovation Ltd) where duplicates will be removed.

### Study Selection

The studies will be selected by 2 independent researchers (ie, the first author, AL, and a research assistant, ML). An initial selection will be made by reading the abstract and title of the article, and then the selected articles will be read in full to retain only those related to the purpose of the study, addressing the research questions, and eligible based on the inclusion and exclusion criteria. In case of disagreement concerning a selection, a third researcher (ie, the second author, VD) will be consulted. As suggested by Levac et al [18], researchers will meet at the beginning, midpoint, and end of the selection process to clarify any difficulties and revise the research strategy. In order to promote transparency, a PRISMA (Preferred Reporting Items for Systematic reviews and Meta-Analyses) diagram will be used to illustrate the study selection process by presenting the articles excluded and the reasons for their exclusion.

### Charting the Data

Data that include authors, year and location of publication, purpose, type of study, population and sample, method, type of intervention and comparison, and intervention components and effects will be extracted to a table. As suggested by Levac et al [18], data from the first 5 papers will be extracted independently by 2 researchers (ie, the first author, AL, and a research assistant, ML) to ensure consistency. Since the purpose of the review is to explore the breadth of knowledge rather than to assess the rigor of the studies identified, the quality of the studies will not be assessed.

### Collating, Summarizing, and Reporting Results

Data will be collected, synthesized, and reported according to the conceptualization of web-based intervention components proposed by Webb et al [13] (ie, the behavior change techniques employed, the delivery modes and theories used, and the effects of the intervention). A thematic analysis, informed by the method used by Paillé and Mucchielli [21], will be conducted to summarize the data by examining the components and effects of the interventions studied. More specifically, themes and subthemes will be identified from the extracted data, based on the various components of the interventions, and categorized under headings representing the different lifestyle habits targeted by the studies. The themes will then be grouped according to their similarity, divergence, complementarity, or recurrence to form thematic clusters (ie, groups of themes with common characteristics). A narrative summary and descriptive tables will be presented to provide an overview of all the components and effects of the interventions studied [18]. The analysis will first be carried out by one author (AL) and validated by the other author (VD). The PRISMA checklist specific to scoping reviews will be used to ensure that all the key items have been reported and in order to promote study replicability [22].

### Consultation

For this step, a focus group will be organized with stakeholders. Since the interventions in the literature will be aimed at older adults, we will ensure that we have their perspective on this issue by consulting 2 people aged 65 years or older who have a lifestyle habit they need to change. In order to be able to deploy this type of intervention in the community, we will ensure that we have a multidisciplinary group of professionals able to provide support to these people as they adopt healthy lifestyle habits. We will, therefore, consult a department head of a primary health care center and health professionals, (ie, a nurse with expertise in preventive health, a nutritionist, and a physiotherapist). The main purpose of this focus group will be to present the preliminary results to these stakeholders and gather their impressions and any new ideas as a way to enrich the results and guide recommendations for future studies.

## Results

We plan to begin the database search in August 2020 and complete the scoping review in October 2020. Upon completion, recommendations will be provided to facilitate integration of web-based interventions into practice to support older adults in healthy lifestyle habits and on the direction of future studies.

## Discussion

### General

In this paper, we have presented the protocol for a scoping review based on 6 steps [18]. The proposed scoping review protocol is innovative in that, to our knowledge, no knowledge synthesis has been conducted on web-based interventions to promote healthy lifestyle behaviors among people aged 65 years and older, nor on their components, theories, and effects on this population. In fact, only 2 systematic reviews [15,16] have been conducted on this subject, but they include primary studies with an average participant age of 50 years or older, such that the results are not specific to a population of older adults. People aged 65 years and older may have different needs, and experience other impacts from interventions aimed at supporting them as they make changes in their lifestyle habits, particularly because of the biological and psychosocial changes associated with aging. This is why such a focus is necessary. Since older adults are a heterogeneous group of people with multiple characteristics and, within this group, different samples of older adults (eg, 85 versus 65 years of age) could present different results, it is important to be attentive to the effects of interventions by age group. Moreover, the 2 knowledge syntheses reviewed focus solely on the quantitative literature, while other types (ie, qualitative) are available in the literature and could greatly enrich our knowledge on this subject. Since the scoping review allows for the inclusion of different types of studies, use of this method appears justified in order to explore the extent of current knowledge on web-based interventions aimed at promoting healthy lifestyles among people aged 65 years and older. The publication of this scoping review protocol also ensures the rigor and the reproducibility of the review, and facilitates transparency regarding the proposed method, which can guide the conduct of new studies on this subject.

Finally, this scoping review will highlight the various web-based interventions aimed at helping older adults adopt healthy lifestyles, as well as the components and effects on people aged 65 years and older. The protocol for this scoping review proposes a novelty—to gather, synthesize and report the data from the literature reviewed according to a conceptualization of web-based interventions proposed by Webb et al [13]. The goal is to identify intervention components that influence the adoption of healthy lifestyle habits among older adults. This conceptualization appears relevant and innovative for this scoping review, since it will guide the synthesis of knowledge by highlighting the behavior change techniques used, modes of intervention delivery, theories used, and effects of web-based interventions in a population of people aged 65 years and older. To our knowledge, this has never been done before. At the end of this scoping review, a synthesis of these elements will be produced and presented in order to systematically identify the various components and effects of the interventions identified. The results of this scoping review may help guide the conduct of new studies on web-based interventions with components adapted to people aged 65 years and older, and encourage the integration of these interventions into practice.

### Strengths

The studies will be selected systematically and independently by 2 researchers (ie, the first author and a research assistant), and the second author will be consulted in case of disagreement, thus ensuring the reliability of the results. The search strategy involves consulting a large number of databases (n=7) as well as grey literature using Google Scholar, OpenGrey, and a manual search of the references of the articles identified, which reduces the risk of articles being omitted. In addition, the study identification process, including inclusion and exclusion criteria, has been explained in detail to ensure reproducibility. The PRISMA checklist specifically for scoping reviews will be used, which will contribute to the transparency and quality of this study [22].

### Limitations

Although this is not the main objective or a necessary step in a scoping review, the quality of the literature will not be assessed in this review, which may raise concerns about the rigor of the literature and affect the generalizability of the results. We will take this limitation into consideration in our analysis of the results and will still critically analyze the literature reviewed. Otherwise, data will be extracted by 2 researchers only for the first 5 articles, whereas independent 2-researcher extraction of data for the entire sample of literature could have enhanced the reliability of the data by avoiding any misinterpretation or omission of information by the authors. Finally, a language restriction (ie, only studies in English and French) will be imposed, which could affect the exhaustiveness of the set of articles identified.

### Conclusion

The purpose of this scoping review will be to explore the extent of the literature on web-based interventions to promote healthy lifestyles among people aged 65 years and older. Given advancements in the use of technologies in the field of health, web-based may be a preferred method for delivering interventions because of its accessibility, especially for a population of older adults, among whom internet use has been growing rapidly in recent years. Given that web-based interventions have the potential to encourage the adoption of healthy lifestyles and thus promote health, it is relevant to examine the components and effects of these interventions among older adults, whom we know are greatly affected by chronic diseases. The results of this scoping review will inform health professionals and interventions developers about the relevant components and effects of web-based interventions in a population of older adults. Knowing this, we can use web-based interventions to promote healthy lifestyles in this population.

## Appendix

Multimedia Appendix 1 Search strategy example.

## Abbreviations

PRISMA: Preferred Reporting Items for Systematic reviews and Meta-Analyses
