# Supplementary material for: Web-Based Interventions to Promote Healthy Lifestyles for Older Adults: Protocol for a Scoping Review
Source: JMIR Res Protoc. 2021 Jan 4;10(1):e23207. doi: 10.2196/23207 (PMC7813627; doi:10.2196/23207)
Supplement: Multimedia Appendix 1 [file resprot_v10i1e23207_app1.docx]

# **Multimedia Appendix 1. Search strategy in CINAHL**

**Search strategy with keywords**

(Aged OR Elder* OR Senior# OR Old N2 person OR Old N2 people OR Older N2 adult# OR Older N2 people OR Aging OR Geriatric# OR Gerontologic*) AND (Web* OR Internet# OR Computer* OR eHeath OR Telemedicine OR Online* OR Telehealth OR telenursing) AND (Lifestyle# OR "Life Style" OR "Lifestyle change OR Habit# OR Behavioral change# OR "Physical activit#" OR Exercice# OR Diet OR Nutrition OR "Weight loss" OR Smoking* OR Smoking N2 Cessation OR tabagism OR Sedentary OR "Sedentary behavior" OR "Sedentary lifestyle" OR Active Lifestyle# OR Alcohol OR "Alcohol drinking" OR "Alcohol abuse" OR Stress OR "Stress management")

**Search strategy with MESH**

( (MH "Aged") OR (MH "Health Services for the Aged") OR (MH "Gerontologic Nursing+") OR (MM "In Old Age") ) AND (MH "Internet") AND ( (MH "Life style change") OR (MH "Health behavior") OR (MH "Life style, Sedentary") OR (MH "Habits+") OR (MH "Behavioral changes") OR (MH "Physical activity") OR (MH "Exercise+") OR (MH "Diet") OR (MH "Nutrition") OR (MH "Weight Loss") OR (MH "Stress") OR (MH "Alcohol drinking") )
